# Comparative Microbiome Study of Mummified Peach Fruits by Metagenomics and Metatranscriptomics

**DOI:** 10.3390/plants9081052

**Published:** 2020-08-18

**Authors:** Yeonhwa Jo, Chang-Gi Back, Hoseong Choi, Won Kyong Cho

**Affiliations:** 1 Research Institute of Agriculture and Life Sciences, College of Agriculture and Life Sciences, Seoul National University, Seoul 08826, Korea; yeonhwajo@gmail.com (Y.J.); bioplanths@gmail.com (H.C.); 2 Horticultural and Herbal Crop Environment Division, National Institute of Horticultural and Herbal Science, RDA, Wanju 55365, Korea; plantdoctor7@korea.kr; 3 Department of Agricultural Biotechnology, College of Agriculture and Life Sciences, Seoul National University, Seoul 08826, Korea

**Keywords:** fungi, bacteria, mycobiome, peach, DNA shotgun sequencing, RNA-sequencing

## Abstract

The dried peach fruits clinging to peach trees or lying on the ground nearby are known as mummified peach fruits. Here, we examined the microbiome communities of three different mummified peach fruits from the nectarine cultivar “Hahong” by DNA- and RNA-sequencing. We found the dominance of *Monilinia fructigena* followed by *Sclerotinia borealis*, *S. sclerotiorum*, and *Botrytis cinerea* in the mummified peach fruits. Moreover, we found a high number of Proteobacteria, including *Frateuria aurantia*, *Neoasaia chiangmaiensis*, *Robbsia andropogonis*, and *Ewingella Americana*. Furthermore, we identified several viruses and viroids. Bacteriophages were identified by DNA- and RNA-sequencing, while viruses and viroids with RNA genomes were identified by only RNA-sequencing. Moreover, we identified a novel mycovirus referred to as *Monilinia* umbra-like virus 1 (MULV1) from *M. fructigena*. Our results revealed the co-inhabitance of fungi and bacteria in the mummified peach fruits, although dominant microorganisms were present. RNA-sequencing revealed that several fungal and bacterial genes were actively transcribed. Comparative analyses suggested that RNA-sequencing provides more detailed information on microbial communities; however, combining DNA- and RNA-sequencing results increased the diversity of microorganisms, suggesting the importance of databases and analysis tools for microbiome studies. Taken together, our study provides a comprehensive overview of microbial communities in mummified peach fruits by DNA shotgun sequencing and RNA-sequencing.

## 1. Introduction

The peach is one of famous stone fruits such as apricot, Japanese mume, plum, and cherry. The peach fruits are widely consumed as fresh or canned peaches. Peach (*Prunus persica* (L.) Batch) is a member of the genus *Prunus* in the family *Rosaceae*. Although a large number of imported fruits have replaced domestic fruits in Korea, the peach fruits cannot be imported due to their short storage period. In Korea, the peach is the third important fruit based on the fruit production and fresh peach fruits are usually produced from July to October. Diverse pathogens and insects are major problems for cultivation of peach trees. Of them, fungal pathogens are regarded as the most severe pathogen, resulting in the loss of quality and quantity of peach production. In particular, it is important to control postharvest fungal diseases in the peach fruit markets [1].

To date, several fungal pathogens in peach fruits have been identified. For example, brown rot (*Monilinia fructicola* and *M. laxa*) [2], grey mold (*Botrytis cinerea*) [3], *Alternaria* spp. [4], *Aspergillus* spp. [5], *Cladosporium* spp. [6], and blue mold (*Penicillium* spp.) [7] have been identified from the peach fruits. In addition, a previous study has reported soil fungal communities in peach orchards revealing the dominance of *Fusarium* species [8]. Of them, the most common fungal disease in peach trees might be brown rot disease caused by *M. fructicola* or *M. laxa*. Brown rot caused by *M. fructicola* causes serious damage on the production of peach fruits and can survive the winter in the mummified peach fruits, producing spores that can infect blossoms and young shoots [9].

The precise diagnostic tools are important to manage fungal diseases. One of conventional methods to diagnose fungal diseases is the culture dependent approach [10]. Cultivable fungi grown on specific medium were isolated followed by identification of fungi based on PCR amplification using fungal specific internal-transcribed-space (ITS) primers. In the case of a culture-independent approach, DNA was directly extracted from samples infected by fungi and used for PCR amplification [11]. Regardless of culture methods, PCR amplification and DNA sequence analyses are now necessary steps to diagnose fungal species. To date, various molecular diagnostic techniques for fungal plant pathogens such as PCR and real-time PCR have been developed [12,13]. For instance, PCR based approaches have been develop to identify *Monilinia* spp. causing brown rot in stone fruit trees [14].

Dried peach fruits clinging to the peach tree or lying on the ground carrying fungi are called as mummified peach fruits. The mummified fruits are a major source for fungal diseases in peach trees. However, little is known for microbial pathogens in mummified peach fruits. In this study, we revealed fungal and bacterial communities in the mummified peach fruits by DNA shotgun sequencing and RNA sequencing.

## 2. Results

### 2.1. Identification of Organisms in the Mummified Peach Fruits by DNA Shotgun Sequencing and RNA-Sequencing

**Figure 1:**
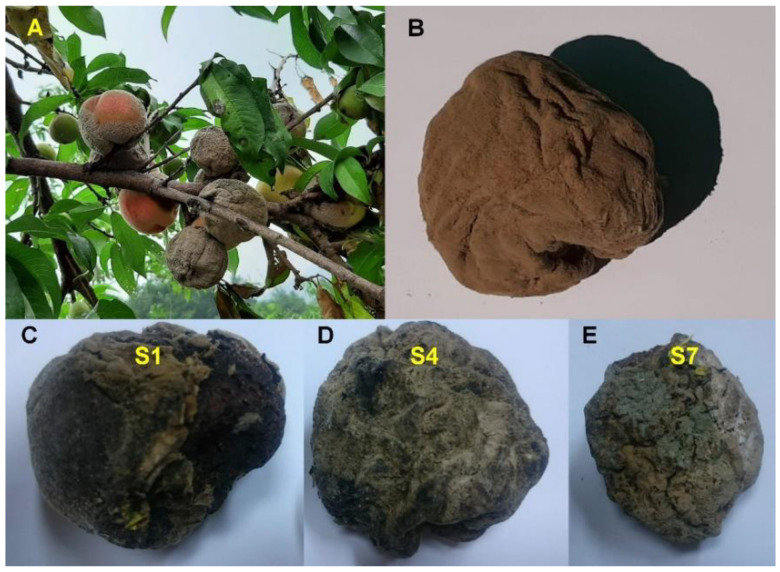
Peach fruits infected by fungi and the mummified peach fruits. (**A**) Peach fruits infected by fungi clinging to the peach branch. (**B**) Brown rot on a peach fruit. Mummified peach fruit samples, S1 (**C**), S4 (**D**), and S7 (**E**) used for next-generation sequencing.

During summer, we can observe many peach fruits infected by fungi clinging to the peach branch in orchards (Figure 1A,B). We collected several mummified peach fruits from the nectarine cultivar “Hahong” in the orchard located in Hoengseong, Korea (Figure 1). Of them, three randomly selected mummified peach fruits were used for total DNA and RNA extraction, respectively (Figure 1C–E). Using extracted DNA and RNA, we generated three DNA and three RNA libraries from the three samples. The libraries were paired-end sequenced using HiSeq2000 system. Numbers of sequence reads in each library were 19,017,846 (D1), 17,076,556 (D4), 16,785,484 (D7), 10,772,266 (R1), 5,681,875 (R4), and 5,702,971 (R7). The raw sequenced were trimmed and clean reads were subjected to metagenome and metatranscriptome analyses.

### 2.2. Taxonomic Classification Using KRAKEN 2 Program

**Figure 2:**
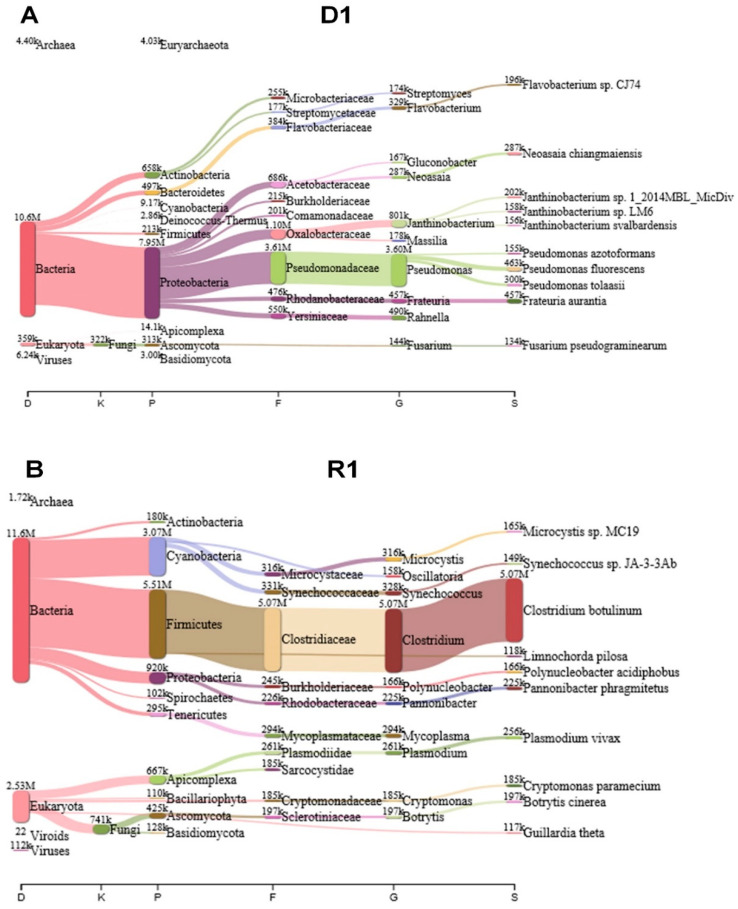
Identification of microorganisms from the D1 and R1 libraries using KRAKEN 2 program. After trimming, clean reads in each library were subjected to a taxonomic classification using KRAKEN 2 against the kraken2-microbial database. The results from the KRAKEN 2 were analyzed and visualized by PAVIAN program. Visualization of identified microorganisms according to taxonomy classification in D1 (**A**) and R1 (**B**) libraries using the Sankey diagram showing the flow of reads from the root of the taxonomy to more specific levels. The width of the flow is proportional to the number of reads. D1 and R1 libraries were derived from the same mummified peach sample.

In order to annotate sequenced reads fast, we initially used KRAKEN 2 program which assigns taxonomic labels of sequencing reads using *k*-mer-based approach with an efficient memory usage [15]. The KRAKEN 2 performed nucleotide search against nucleotide database (kraken2-microbial database) (September 2018, 30GB). The KRAKEN 2 programs revealed diverse microorganisms including fungi, bacteria, viruses, and viroids in the mummified peach fruits. For example, several proteobacteria such as *Pseudomonas* spp. were dominantly identified in D1 library (Figure 2A) while a large number of reads associated with *Clostridium botulinum* were identified in R1 library (Figure 2B). As compared to D1 and R1 libraries, other four libraries contain a large number of reads associated with *Botrytis cinerea* followed by *Fusarium pseudograminearum* (Figures S1 and S2). In addition, several proteobacteria such as *Pantoea ananatis*, *Aspergillus oryzae*, *Pseudomonas tolaasii*, and *Kosakonia cowanii* were also identified in D4, R4, D7, and R7 libraries (Figures S1 and S2). In D1 and R1 library, bacteria; reads were dominant whereas fungal reads were dominant in D4, R4, D7, and R7 libraries. However, the fungal metagenome and metatranscriptome results from KRAKEN2 were not consistent with other experimental data demonstrating that mummified peach fruits should be dominated by *Monilinia* species. In fact, the microbial database used for KRANEN 2 did not contain enough sequence data for plant fungal metagenome and metatranscriptome analyses. Instead of using KRAKEN 2, we carried out BLASTX search against NCBI’s non-redundant (NR) protein database for sequence annotation using DIAMOND program with default parameters [16]. The BLASTX search was much slower than KRAKEN 2; however, the NR protein database contains a large number of sequence data including fungi and BLASTX using translated search provided accuracy for taxonomic classification. The DIAMOND BLASTX results were imported into MEGAN6 for taxonomy classification [17].

### 2.3. Classification of Identified Organisms on the Three Mummified Peach Fruits According to Domain

**Figure 3:**
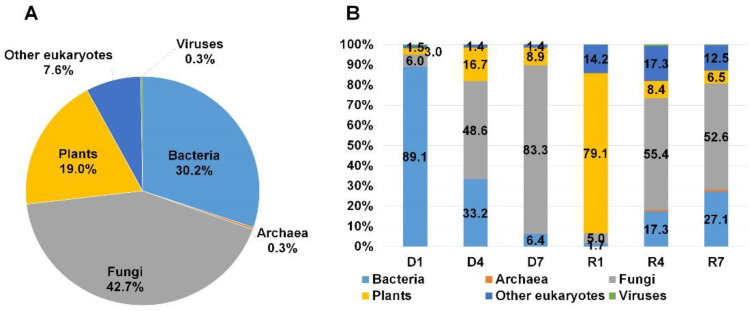
Classification of identified organisms from three mummified peach fruits according to domain. (**A**) Proportion of identified organisms after combining reads from all six different libraries (three DNA and three RNA libraries) based on the number of assigned reads. (**B**) Proportion of identified organisms in each library based on the number of assigned reads. To calculate proportion of identified organisms, we used number of reads assigned to individual organisms identified by MEGAN6 program. The number of reads was not normalized. Three DNA libraries (D1 to D7) and three RNA libraries (R1 to R7) were prepared from S1, S4, and S7 mummified peach fruit samples.

In order to observe the taxonomy overview of identified organisms, we combined all annotation results from six libraries according to domain using MEGAN6 program. As we expected, the reads associated with fungi (42.7%) were abundantly present followed by bacteria (30.2%) and plants (19%) (Figure 3A). In addition, we identified several reads associated with other eukaryotes (7.6%), archaea (0.3%), and viruses (0.3%). Next, we examined the proportion of identified organisms in each library according to domain (Figure 3B). Interestingly, D1 and R1 libraries were derived from the identical S1 sample, bacterial reads (89.1%) were dominantly present in D1 library while reads associated with plants (79.1%) were dominantly present in R1. By contrast, fungal reads were abundantly identified in other four libraries followed by bacterial reads. Of six libraries, the proportion of fungal reads (83.3%) was very high among six libraries.

### 2.4. Classification of Identified Fungi According to Fungal Taxonomy

**Figure 4:**
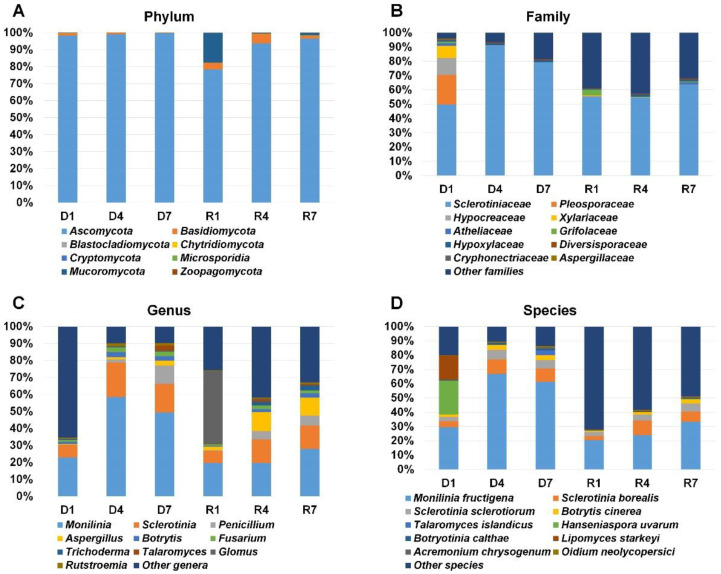
Taxonomy classification of identified fungi from six different libraries. Results of BLASTX using Diamond were subjected to MEGAN6 to classify taxonomy of identified organisms according to phylum (**A**), family (**B**), genus (**C**), and species (**D**). For simplicity, only a proportion of representative fungal taxonomy was visualized.

Main purpose of this study was the identification of fungi in the mummified peach fruits. Therefore, we first collected taxonomy information for fungi. Based on the phylum, we identified eight different phyla in six libraries (Table S1 and Figure 4A). Fungi belonging to the phylum *Ascomycota* were dominantly present in all six libraries (78.2% to 99.08%). In R1 library, *Ascomycota* (78.24%) was the dominant phylum followed by *Mucoromycota* (17.61%). Fungi in the phylum *Basidomycota* were identified in all six libraries. In particular, the proportion of *Basidomycota* in R1 and R4 was relatively higher than in other four libraries. According to the fungal family, the family *Sclerotiniaceae* was the most dominant fungal family in all libraries (Figure 4B). The D4 library (91%) has high amount of fungi in the family *Sclerotiniaceae* whereas 49.7% of fungi in the D1 library belongs to the family *Sclerotiniaceae*. Among six libraries, diverse fungal families were identified from D1 library. For example, *Sclerotiniaceae* (49.7%) was the dominant family followed by *Pleosporaceae* (20.7%), *Hypocreaceae* (11.8%), *Xylariaceae* (8.4%), *Atheliaceae* (2%), and *Grifolaceae* (1.1%) in D1 library. Based on the genus, *Monilinia* was the most frequently identified genus in all libraries except R1 (Figure 4C). The proportion of *Monilinia* in each library ranged from 19.5% (R1) to 58.5% (D4). The *Sclerotinia* was the second frequently identified genus in most libraries. Interestingly, the proportion for the genus *Glomus* (42.8%) was the highest in R1 as compared to other libraries. The genus *Aspergillus* was identified in all six libraries and its proportion was very high in R4 and R7 libraries. In addition, we identified fungi belonging to the genus *Penicillium*. We identified various fungal species (Figure 4D). Of them, *Monilinia fructigena* was the most dominant species in all six libraries. The proportion of *Monilinia fructigena* was higher in three DNA libraries as compared to three RNA libraries. *Sclerotinia borealis* and *Sclerotinia sclerotiorum* were also frequently identified in most libraries. In D1 library, the proportion of *Hanseniaspora uvarum* and *Lipomyces starkeyi* was relatively high compared to other libraries.

### 2.5. Major Fungal Species in Each Library

**Figure 5:**
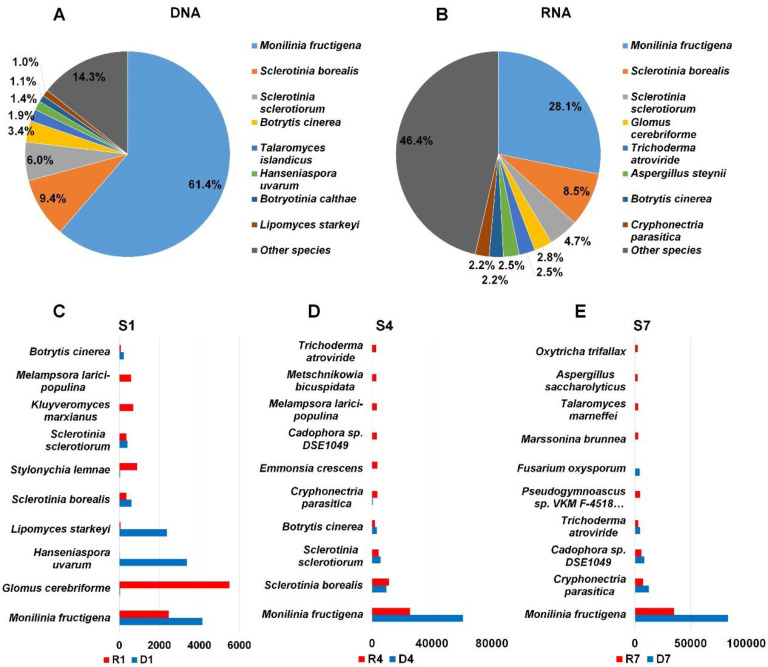
Proportion of major fungal species identified from three DNA and three RNA libraries. The proportion of major fungal species identified from three DNA (**A**) and RNA (**B**) libraries. The results of fungal species in each library were combined according to DNA and RNA libraries. The top ten fungal species in each sample, S1 (**C**), S4 (**D**), and S7 (**E**). Number indicates the number of raw sequence reads associated with corresponding fungal species. Red and blue colors indicate RNA and DNA libraries.

It might be of interest to compare the proportion of identified fungal species between DNA and RNA library although identical sample was used for nucleic acid extraction. To simplify the comparison, we combined all fungal taxonomy information according to DNA and RNA. By combining all three DNA libraries, we found that 61.4% of fungal reads in DNA libraries were associated with *Monilinia fructigena* causing brown rot disease (Figure 5A). Similarly, *M. fructigena* (28.1%) was the dominant fungal species in RNA libraries (Figure 5B). The second frequently identified fungal species in DNA libraries was *Sclerotinia borealis* (9.4%) followed by *S. sclerotiorum* (9.4%), *Botrytis cinerea* (3.4%), and *Talaromyces islandicus* (1.9%) (Figure 4A). In RNA libraries, *S. borealis* was again the second dominant fungal species followed by *S. sclerotiorum* (4.7%), *Glomus cerebriforme* (2.8%), and *Trichoderma atroviride* (2.5%) (Figure 4B). The proportion for other fungal species in DNA libraries (14.3%) was lower than that in RNA libraries (46.4%).

Next, we selected top ten fungal species in sample based on assigned read number. In S1 sample, the number of reads associated with *M. fructigena* was very high in both DNA (4161 reads) and RNA (2488 reads) (Figure 5C). Interestingly, a large number of reads associated with *G. cerebriforme* was identified from the RNA library (5515 reads) whereas only 38 reads for *G. cerebriforme* were identified in the DNA library. Similarly, the number of reads associated with *Hanseniaspora uvarum* (3379 reads) and *Lipomyces starkeyi* (2384 reads) in the DNA library was much higher than that in the RNA library (Figure 5C). In both S4 and S7 libraries, the number of reads associated with *M. fructigena* was two times higher in DNA libraries than that in RNA libraries. Several additional fungal species such as *Cryphonectria parasitica*, *Emmonsia crescens*, *Cadophora* sp. DSE1049, *Melampsora larici-populina*, *Metschnikowia bicuspidate*, and *Trichoderma atroviride* were identified from S4 sample (Figure 5D). In S7 library, we identified *Pseudogymnoascus* sp., *Fusarium oxysporum*, *Marssonina brunnea*, *Talaromyces marneffei*, *Aspergillus saccharolyticus*, and *Oxytricha trifallax* (Figure 5E).

### 2.6. Comparison of Identified Fungal Species among Different Libraries and Samples

**Figure 6:**
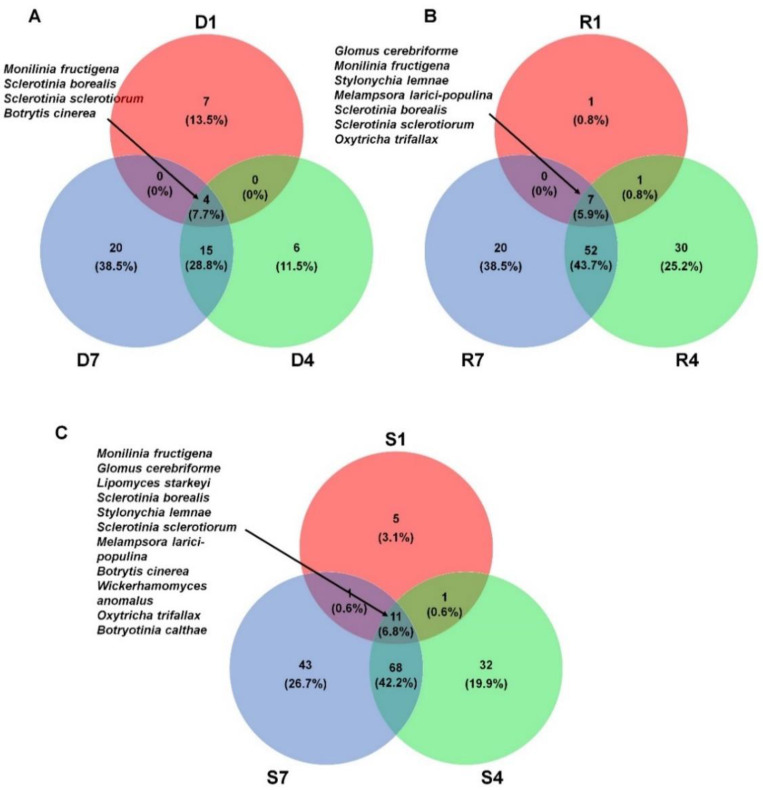
Comparison of identified fungal species in three different samples. Venn diagram showing commonly identified fungal species among three different DNA libraries (**A**), three different RNA libraries (**B**), and three different samples (**C**). For comparison, we only selected fungal species with the number of reads more than 100 in each sample. The numbers indicate the number of identified fungal species and the proportion in each sample.

We compared the list of identified fungal species among different libraries. For that, we selected fungal species in each library based on the number of assigned read more than 100. As a result, we identified 11, 25, and 39 species from D1, D4, and D7 library, respectively (Figure 6A). Four fungal species including *M. fructigena*, *S. borealis*, *S. sclerotiorum*, and *B. cinerea* were commonly identified in the three DNA libraries. In addition, there were 19 fungal species commonly identified in both D4 and D7 libraries. Each DNA library has unique fungal species such as seven species (D1), six species (D4), and 20 species (D7). A total of seven fungal species including *G. cerebriforme*, *M. fructigena*, *Stylonychia lemnae*, *Melampsora larici-populina*, *S. borealis*, *S sclerotiorum*, and *Oxytricha trifallax* were commonly identified in three RNA libraries (Figure 6B). The number of commonly identified fungal species (59 species) between R4 and R7 was very high. Except R1 (one species), R4 (30 species) and R7 (20 species) have many library specific fungal species. Next, we compared the list of identified fungal species among three different samples (Figure 6C). A total of 11 fungal species were commonly identified in all three samples. The number of sample specific fungal species was five species (S1), 32 species (S4), and 43 species (S7). Again, many fungal species (79 species) were commonly identified in both S4 and S7 samples.

### 2.7. Classification of Identified Bacteria According to Bacterial Taxonomy

**Figure 7:**
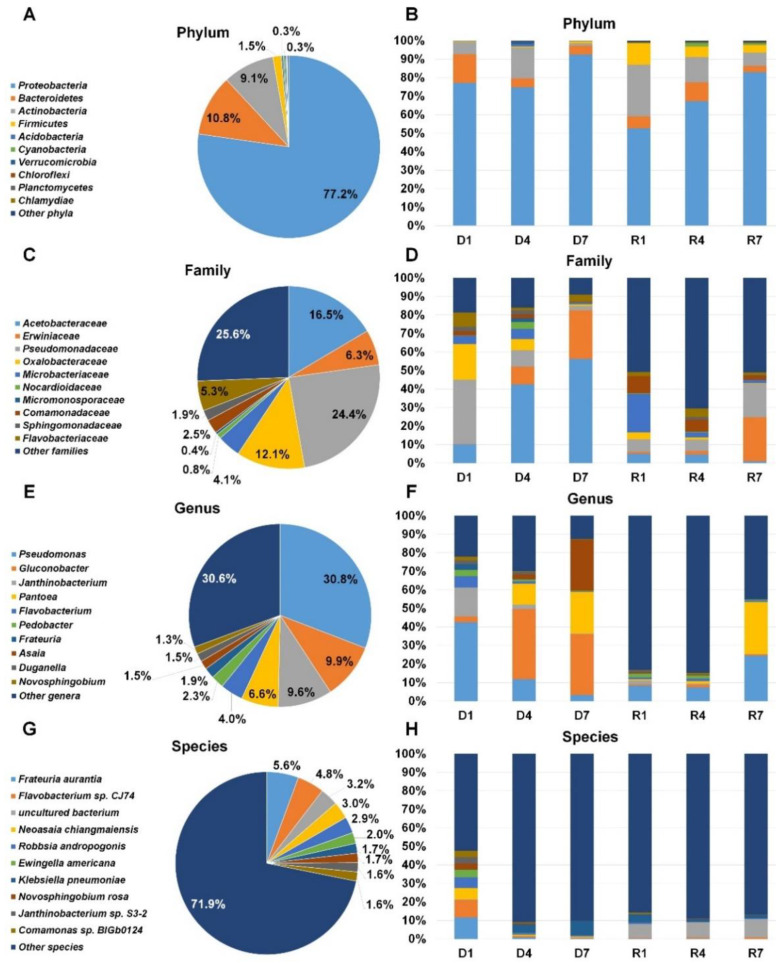
Taxonomy classification of identified bacteria from six different libraries. Results of BLASTX using DIAMOND were subjected to MEGAN6 to classify taxonomy of identified organisms according to phylum (**A**), family (**C**), genus (**E**), and species (**G**). For simplicity, only proportion of representative bacteria taxonomy was visualized. The proportion of identified major bacterial taxonomy according to phylum (**B**), family (**D**), genus (**F**), and species (**H**).

Not only fungi but also diverse bacteria were identified by DNA shotgun sequencing and RNA-sequencing (Table S2 and Figure 7). According to phylum, *Proteobacteria* (77.2%) was the dominant bacterial phylum followed by *Bacteroidetes* (10.8%), *Actinobacteria* (9.1%), and *Firmicutes* (1.5%) after combining all reads associated bacteria (Figure 7A). The proportion of *Proteobacteria* in each library ranged from 52.7% (R1) to 92.4% (D7). *Actinobacteria* was the second dominant bacterial phylum in D4 (16.4%), R1 (27.7%), R4 (13.6%), and R7 (7.2%) (Figure 7B). *Bacteroidetes* was the second dominant bacterial phylum in D1 (15.4%) and D7 (4.7%). After combining all bacterial reads according to family, *Pseudomonadaceae* (24.4%) was the dominant family followed by *Acetobacteraceae* (16.5%), *Oxalobacteraceae* (12.1%), *Erwiniaceae* (6.3%), and *Flavobacteriaceae* (5.3%) (Figure 7C). The *Pseudomonadaceae* was the dominant family only in D1 library (34.8%) whereas *Acetobacteraceae* was the dominant family in D4 (42.5%) and D7 (56.1%) libraries (Figure 7D). Interestingly, three different bacterial families were dominant in R1 (*Microbacteriaceae*), R4 (*Comamonadaceae*), and R7 (*Erwiniaceae*). *Pseudomonas* (30.8%), *Gluconobacter* (9.9%), *Janthinobacterium* (9.6%), *Pantoea* (6.6%), and *Flavobacterium* (4.0%) were major bacterial genera after combining all identified reads associated with bacterial genera (Figure 7E). In D1 library, *Pseudomonas* (42.4%) was the dominant followed by *Janthinobacterium* (15.5%) (Figure 7F). The genus *Guconobacter* was dominantly present in D4 and D7 libraries. In D7 library, *Guconobacter* (32.6%), *Asaia* (27.5%), and *Pantoea* (22.4%) were major genera. In R7, *Pseudomonas* (24.5%) and *Pantoea* (28.2%) were major genera. At the species level, numerous bacterial species were identified. Of them, the representative bacterial species were *Frateuria aurantia*, *Flavobacterium* sp. CJ74, uncultured bacterium, *Neoasaia chiangmaiensis*, and *Robbsia andropogonis* in all six libraries (Figure 7G). In D1, *Frateuria aurantia* was the main bacteria species whereas *Klebsiella pneumonia* was dominant bacteria species in D4 and D7 (Figure 7H). Uncultured bacterium was dominant in three RNA libraries.

### 2.8. Identification of Viruses from Six Libraries

BLASTX against the NR database revealed several virus-associated reads from six libraries (Table S3). From the three DNA libraries, several bacteriophages with double-stranded DNA genomes were identified. For example, *Pseudomonas* virus Andromeda, *Pseudomonas* virus Bf7, and acinetobacter phage ABPH49 were identified from the D1 library, while two bacteriophages, *Pseudomonas* phage Phabio and Caudovirales sp., were identified from the D4 library. In the D7 library, *Pseudomonas* phage OBP was identified. By contrast, DNA viruses as well as RNA viruses were identified from the three RNA libraries. In the R1 library, *Sclerotinia sclerotiorum* umbra-like virus 3 (SsULV3), a mycovirus infecting fungi with the dsRNA genome, was identified [18]. In the R4 library, many virus-associated reads showed sequence similarity to *S. sclerotiorum* ourmia-like virus 1 (SsOLV1), *S. sclerotiorum* ourmia-like virus 2 (SsOLV2), and *S. sclerotiorum* umbra-like virus 3 (SsULV3). In addition, we identified several plant viruses infecting peach trees, including Asian prunus virus 2 (APV2) and apple chlorotic leaf spot virus (ACLSV). In the R7 library, *Pseudomonas* phage OBP was the most abundant virus followed by SsOLV1 and SsOLV2.

### 2.9. Identification of Plant Viruses, Viroids and a Novel Mycovirus

**Figure 8:**
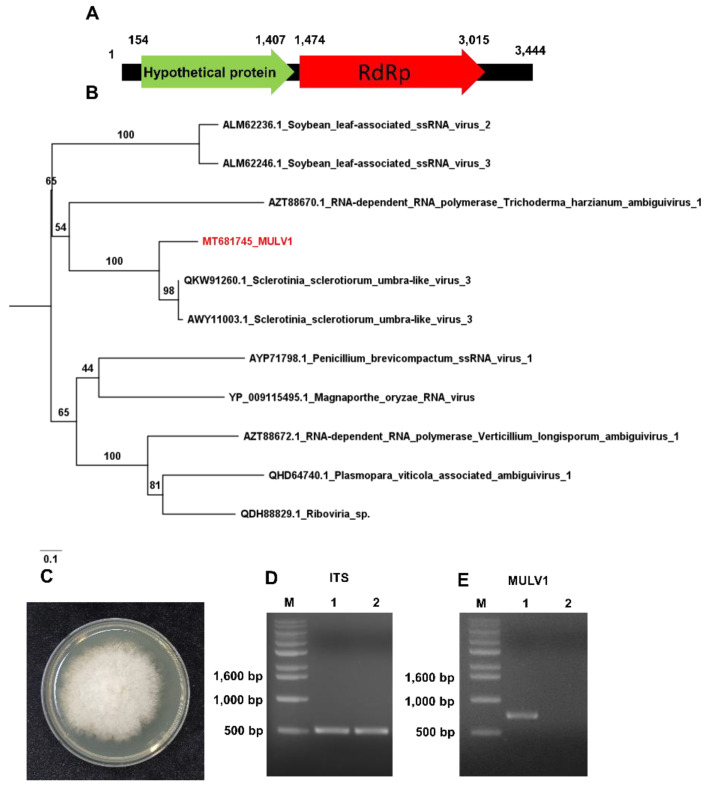
Genome organization and phylogenetic relationship of novel mycovirus (MULV1). (**A**) Genome organization of MULV1. Numbers indicate positions of open reading frames (ORFs). (**B**) Phylogenetic relationship of MULV1 and closely related viruses. The phylogenetic tree was inferred by IQ-TREE with the maximum likelihood method and 1000 bootstrap iterations. (**C**) Isolated *Monilinia fructigena* from the mummified peach fruit (S4 sample) grown on potato dextrose agarose (PDA) medium. (**D**) Identification of *M. fructigena* by PCR using internal transcribed spacer (ITS) primers. M indicates DNA ladder. 1 and 2 indicate *Monilinia* species from the mummified peach fruit (S4 sample) and other peach fruit, respectively. (**E**) RT-PCR results using MULV1 specific primers. The amplicon size is 737 bp.

To determine the viral sequences of the identified RNA viruses in detail, we conducted de novo transcriptome assembly for the three RNA libraries using the Trinity program. The assembled contigs for each library were subjected to BLASTN search against the viral genome database. After removing non-viral sequences, we identified several virus- and viroid-associated contigs. For example, hop stunt viroid (HSVd) was identified from the R1 and R4 libraries, while peach latent viroid (PLMVd) was identified from all three RNA libraries (Table 1). Consistent with the BLASTX results against NR, ACLSV was identified from the R4 library, while APV2 and Asian prunus virus 3 (APV3) were identified from the R4 library. Moreover, we assembled complete genomes for HSVd (297 nt) and PLMVd (337 nt). Furthermore, we obtained two contigs associated with *Sclerotinia sclerotiorum* umbra-like virus 3 (SsULV3) from the R1 and R4 libraries. Based on the assembled viral sequences and RT-PCR, we obtained the complete genome sequence for a novel mycovirus referred to as *Monilinia* umbra-like virus 1 (MULV1) isolate Won 3,444 bp in length. We found that all partial sequences associated with SsOLV1–SsOLV3 were derived from MULV. The genome sequence of MULV1 showed sequence similarity to SsULV3 with 70% coverage and 78.22% nucleotide identity. We found that MULV1 encodes two ORFs, ORF1 (position 154–1407) and ORF2 (1474–3015) (Figure 8A). The BLASTP results showed that a hypothetical protein of MULV1 showed sequence similarity to a hypothetical protein of SsULV3 (AWY11004.1) with 98% coverage and 65.86% protein identity, while ORF2 of MULV1 showed sequence similarity to the RNA dependent RNA polymerase (RdRp) of SsULV3 (QKW91260.1) with 99% coverage and 81.05% protein identity. A phylogenetic tree created using RdRp amino acid sequences displayed that MULV1 was in the same clade as SsULV3 (Figure 8B).

To confirm the infection of MULV1 in the *M. fructigena*, we cultured fungi from the mummified peach fruit (S4 sample). Of the recovered fungi on PDA medium, we identified *M. fructigena* by PCR using internal transcribed spacer (ITS) primers followed by Sanger-sequencing (Figure 8C,D). Using MULV1 specific primers, we carried out RT-PCR using total RNAs of the *M. fructigena* from the mummified peach fruit (S4 sample) and other peach fruit. RT-PCR and Sanger-sequencing results confirmed the infection of MULV1 in *Monilinia* species derived from the mummified peach fruit and not the other peach fruit.

## 3. Discussion

Recently, the rapid development of next-generation sequencing techniques facilitates microbiome associated studies. In general, PCR amplification of marker DNA regions such as 16S ribosomal RNA for bacteria and ITS region for fungi followed by NGS might be the most efficient way to reveal microbial communities in a given sample [19]. Amplicon based microbiome studies can reveal only known microorganisms and single PCR reaction can amplify only partial sequence of bacteria or fungi not both. However, DNA shotgun sequencing or RNA-sequencing has many advantages than the 16S amplicon method such as increased detection for microorganism species and diversity [20]. In addition, it can identify uncultivable microorganism.

In this study, we identified a large number of fungal and bacterial species in the mummified peach fruits by DNA shotgun sequencing and RNA-sequencing. To increase accuracy for microorganism identification, we applied stringent detection limit (the number of reads for the identification of a single species more than 100 reads) as suggested by a recent study [21] and other microorganism species with a lower abundance were removed. As a result, we identified at least 197 fungal and 283 bacterial species in the mummified peach fruits. The most frequently identified fungal species were *M. fructigena*, *S. borealis*, *S. sclerotiorum*, and *B. cinerea*. As we expected, *M. fructigena* species were dominantly identified in three peach samples. It has already known that diverse *Monilinia* species causing brown rot disease leads to the dramatic losses of post harvested peach fruits [22]. Geographically, *M. fructicola* is a dominant species in America while *M. fructigena* and *M. laxa* are frequently identified in Europe [23]. Previously, infection of *M. fructicola* in different stone fruits in Korea has been reported [24]; however, infection of other *Monilinia* species in stone fruits has not been reported in our knowledge. This is the first report of *M. fructigena* in stone fruits including peach in Korea. We only collected peach samples in an orchard, therefore, the occurrence of *M. fructigena* in different regions in Korea should be further examined. Moreover, the orchard in which we collected samples has many different stone fruits including peach, Japanese plum, apricot, and cherry. Therefore, the infection of *M. fructigena* in other stone fruits in the same orchard might be very high.

In addition, we analyzed our sequence data using two different approaches, KRAKEN 2 and DIAMOND. KRAKEN 2 was a fast and efficient program for microbiome study such as bacteria [15]. By contrast, DIAMOND program based on BLASTX search took longer time, it revealed better detailed taxonomic classification for fungal communities. In general, the translated search using a nucleotide sequence to search a protein sequence database showed much higher accuracy than other nucleotide searches [25]. The BLASTX using DIAMOND is about 2500 times faster than a normal BLASTX and overcomes the disadvantage of BLASTX search requiring a longer running time [16]. For microbiome study, not only data analysis programs but also databases are important factors for successful identification of microorganisms in a given sample [26]. As compared to bacteria, the limitation of fungal genomic data was a major obstacle for fungal identification. For example, using the microorganism database containing bacteria, fungi, and viruses for KRAKEN 2, only *B. cinerea* was identified in this study. By contrast, we identified several important fungal species using NR database. This result clearly demonstrates the importance of database for microbiome analyses using next-generation sequencing. Although we identified several fungal species, it is possible that there might be several unknown fungal species from the mummified peach fruits. Fortunately, genome and transcriptome data for several *Monilinia* species causing brown rot disease in stone fruits and pome are currently available. For instance, genomes of *M. fructigena* [27] and *M. fructicola* [28]. Moreover, a recent study reported draft genomes of four different *Monilinia* species [29]. In addition, transcriptomes of three *Monilinia* species have recently published [30]. Furthermore, PCR based markers to distinguish different *Monilinia* species have been developed [31,32].

In this study, we analyzed fungal and bacterial communities in the mummified peach fruits by two different approaches including DNA shotgun sequencing (Metagenomics) and RNA-sequencing with poly(A) selection (Metatranscriptomics). Although shotgun metagenomics is an efficient way to identify a wide range of species, but it has not been well used for fungal identification. A previous study has successfully identified fungi in the animal metagenomes containing 71 fungal species from 39 datasets using newly developed FindFungi program [33]. Other study demonstrated that DNA shotgun sequencing was superior to detect lichen fungal diversity in environmental samples as compared to amplicon based methods [34]. RNA-sequencing with poly(A) selection cannot be used for the majority of bacteria, and many viruses without poly(A) tails cannot be selected by oligo dT for mRNA preparation. Therefore, a fewer number reads associated with bacteria were identified by RNA-sequencing as compared to DNA-sequencing. If we prepare RNA libraries using total RNAs instead of mRNAs, the proportion of bacteria-associated reads by RNA-sequencing might be increased.

We identified several viruses and viroids by DNA- and RNA-sequencing. The most abundant viruses in the DNA libraries were bacteriophages with double-stranded DNA genomes; however, bacteriophages were identified by RNA-sequencing, which can reveal phage transcripts. Although our experiment was not designed to capture viral RNA genomes, we identified viruses with RNA genomes, such as plant viruses and viroids, by RNA-sequencing with poly(A) selection. Our previous peach virome study using RNA-sequencing with mRNA library also demonstrated that different types of viral genomes, regardless of the presence of poly-A, can be identified by RNA-sequencing with poly(A) selection [35]. Based on the previous report showing widespread 3′ uridylation in eukaryotic viruses [36], we carefully supposed that some (U)-rich region in the viruses without poly(A) might be selected by oligo dT. Moreover, it is noteworthy that the RNA-sequencing could detect several plant viruses and two viroids in the mummified fruits. Our result is consistent with a previous report identifying PLMVd from 50-year-old dried leaf material by RT-PCR (Guy 2013). Thus, dried plant materials could be a reservoir of RNA viruses and viroids in orchards. RNA-sequencing identified a novel mycovirus infecting *Monilinia* species. We obtained the complete genome of MULV1 from the R1 and R4 libraries, which were severely infected by *M. fructigena* species. The phylogenetic tree and BLAST result indicated that MULV1 is closely related with the known SsULV3 from *S. sclerotiorum*. Both *M. fructigena* and *S. sclerotiorum* belong to the family *Sclerotiniaceae*. This result suggests that the two virus genomes might have diverged from one fungal host.

In this study, we used the identical sample for DNA and RNA extraction; however, DNA- and RNA-sequencing results were different from each other. For example, there were some bias such as *Lipomyces starkeyi*, *Hanseniaspora uvarum*, and *G. cerebriforme* between DNA and RNA sequencing results. It was quite hard for us to find correlation between DNA and RNA sequencing. We suppose that the experimental design and sample size might not be appropriate to reveal correlation between DNA- and RNA-sequencing. In general, DNA-sequencing results provide the presence of microorganisms while RNA-sequencing with poly(A) selection results indicate the expression of genes for identified organisms as described previously [37]. Interestingly, we discovered high abundance of actively transcribed fungal genes in the mummified peach fruits by RNA-sequencing, suggesting that they are potential threats for peach brown rot disease. Based on our results, RNA-sequencing allowed us to identify a high number of diverse fungal species than DNA-sequencing. A previous study demonstrated the advantage of metatranscriptome to detect fungi with low abundance from soil samples [38]. Another study also recommended a meta-total RNA sequencing (MeTRS) method using total RNAs for the study revealing complex communities consisted of bacteria, fungi, and other microbes [39]. Thus, it might be for us better to use total RNAs instead of mRNAs for the microbiome study. It was evident that using both sequencing techniques increase the diversity of microorganism identification by complementing the disadvantage of individual technique. For instance, commonly identified fungal species were only four (DNA sequencing) and seven (RNA-sequencing) whereas 11 fungal species were commonly identified by combining both results. The main two purposes of this study were identification of fungal and bacterial species as well as to obtain DNA- and RNA-sequences associated with identified organisms. The DNA- and RNA-sequencing data provides sequence information which can be usefully applied for the development of molecular diagnostics of identified fungi and bacteria.

## 4. Materials and Methods

### 4.1. Sample Collection

We collected mummified peach fruits in an orchard located in Hoengseong, Gangwon province in February 2019. Some mummified peach fruits were hanged on the branch of peach trees; however, we randomly sampled the mummified peach fruits on the field. The peach trees for the mummified peach fruits were grown in proximity to each other (2-m × 2-m). All mummified peach fruits in this study were derived from the cultivar “Hahong”, which is a kind of nectarine with smoothy skin. The mummified peach fruits were collected from an area with a radius of 3 m. Only specific peach cultivars producing relatively small size of peach fruits had mummified peach fruits. The collected individual sample was kept in a plastic bag and frozen in the presence of liquid nitrogen.

### 4.2. Extraction of Nucleic Acids from the Mummified Peach Fruits

The mummified peach fruits were well dried. The frozen samples were too hard to grind, we crushed samples using a hammer. After that, samples were ground in the presence of liquid nitrogen by using mortar and pestle. The fine powder was used for nucleic acid extraction. Genomic DNA was extracted by using Qiagen DNeasy Plant Mini Kit (Qiagen, Hilden, Germany) according to manufacturer’s instructions. Total RNA was extracted by using Fruit-mate for RNA Purification (Takara, Shiga, Japan) and the RNeasy Plant Mini Kit for RNA extraction (Qiagen), following the manufacturers’ instructions. We checked the quality and quantity of extracted nucleic acids by agarose gel electrophoresis and NanoDrop Spectrophotometers (Thermo Fisher Scientific, Waltham, MA, USA). Three samples (S1, S4 and S7) showing high quality of DNA and RNA were further used for library preparation.

### 4.3. Library Preparation and Next-Generation Sequencing

From three samples, we prepared three different DNA libraries using NEBNext Ultra II DNA Library Prep with Sample Purification Beads (NEB, Ipswich, MA, USA) following manufacturer’s instruction. The extracted total RNAs were subjected for mRNA extraction based on using the NEBNext Poly(A) mRNA Magnetic Isolation Module (NEB) according to the manufacturer’s instructions. We used the NEBNext Ultra RNA Library Prep Kit for Illumina (NEB) in order to prepare three different RNA libraries using following the manufacturer’s instructions. Individual DNA and RNA libraries were indexed using NEBNext Multiplex Oligos for Illumina (Index Primers Set 1) (NEB). We measured the quality of the generated libraries by using a 2100 Bioanalyzer (Agilent, Santa Clara, USA). A total of six libraries composed of three DNA and three RNA libraries were paired-end (2 × 100 bp) sequenced by Macrogen Co. (Seoul, South Korea) using the HiSeq 2000 platform. We deposited all raw sequences in the SRA database in NCBI with respective accession numbers: SRR11531522 (D1), SRR11531521 (D4), SRR11531520 (D7), SRR11531519 (R1), SRR11531518 (R4), and SRR11531523 (R7) under BioProject PRJNA624910.

### 4.4. Bioinformatic Analyses

The obtained raw data were trimmed using the PRINSEQ program with the quality score 20 [40]. We did not remove sequence copies. The clean reads were subjected to taxonomic classification using two different approaches. The fist was KRAKEN 2 providing a fast taxonomic classification of metagenomics sequence data [15]. The kraken2-microbial database (September 2018, 30GB) was used as a reference database for KRAKEN 2. We conducted KRAKEN 2 with default parameters. The KRAKEN report file for each library was analyzed by PAVIAN program. The second approach was BLASTX search against NCBI’s non-redundant (NR) protein database using DAIMOND program [16]. The BLASTX results were applied into MEGAN6 program [17] to annotate taxonomy of individual sequences. The KRAKEN 2 conducts nucleotide search against nucleotide database while DIAMOND using BLASTX conducts translated search using nucleotide sequences against protein database.

### 4.5. De Novo Transcriptome Assembly and Virus Identification

To obtain complete viral genome sequences, de novo transcriptome assemble was carried out using the Trinity program with default parameters as described previously [41]. The assembled contigs were subjected to BLASTN search with E-value 1e-10 as a cutoff against the viral database derived from the NCBI. All virus-associated contigs were subjected to BLASTX search against the NCBI non-redundant (NR) protein database to remove non-viral sequences. We assembled complete genome sequences for two viroids and a novel mycovirus. To calculate number of virus-associated reads and coverage, raw sequence reads were mapped on the identified virus reference genome sequences using a Burrows–Wheeler Aligner (BWA) program with default parameters [42]. From the mapped SAM file, we calculated coverage and the number of virus-associated reads using pileup.sh implemented in BBMap.

### 4.6. RT-PCR and Sanger-Sequencing

To confirm the genome of a novel mycovirus, we designed primer-pairs, p1r-4121-273F1 (5′-CAAGCAAGGTAAGCTACTGCCTCA-3′) and p1r-4121-3716R1 (5′-GTCATTCCTACCCTGCGCCC-3′) based on the assembled mycovirus genome sequence. cDNA was synthesized using the SuperScript III Reverse Transcriptase (Invitrogen, Carlsbad, CA, USA). Using the synthesized cDNA as the template, PCR was conducted using EX-Taq polymerase (Takara Korea Biomedical Inc., Seoul, Korea). The amplified PCR product was cloned to pGEM^®^-T Easy Vector (Promega, Madison, WI, USA). Finally, we determined complete genome sequence of the novel mycovirus by Sanger-sequencing.

### 4.7. Construction of Phylogenetic Tree for the Novel Virus

We retrieved top 10 RdRp protein sequences showing sequence similarity to the novel mycovirus. A total of 11 RdRp sequences were aligned by the MAFFT program with the L-INS-I option [43]. We trimmed aligned protein sequences using the trimAL program with the automated 1 option [44]. Trimmed nucleotide sequences were subjected to ModelFinder implemented in the IQ-TREE program to select the best-fit model according to the Bayesian information criterion (BIC) [45]. We generated the phylogenetic tree for RdRp using IQ-TREE with the selected best-fit model (LG+F+I+G4), Ultrafast Bootstrap with 1000 iterations [46,47], and the SH-aLRT branch test [48]. We visualized the generated phylogenetic tree using the FigTree (version 1.4.4) program (https://github.com/rambaut/figtree/releases).

### 4.8. Confirmation of the Host for the Novel Mycovirus

To confirm that the identified mycovirus was derived from the fungal host, we cultured fungi from the mummified peach samples on potato dextrose agar (PDA) medium. Of the recovered fungi, we identified *Monilinia* species by PCR using ITS primers (ITS1 5′-TCCGTAGGTGAACCTGCGG-3′ and ITS4 5′-TCCTCCGCTTATTGATATGC-3′) followed by Sanger-sequencing. Total RNAs were extracted from mycelia after 3 days of incubation on PDA medium for the cultured *Monilinia* species. Based on RdRp sequence of MULV1, we designed primers (MULV1_forward 5′-AATGTGGGGCCAAAGTGGATG-3′ and MULV1_reverse 5′-GTGGCGCCAGACAACACCTT-3′) for RT-PCR. Using the extracted total RNAs, we carried out RT-PCR using SolGent DiaStar One-Step RT-PCR kit (SolGent, Daejeon, Korea) according to manufacturer’s instruction.

## 5. Conclusions

Here, we carried out DNA shotgun sequencing and RNA sequencing to reveal microbiome associated with the mummified peach fruits. Combining results from both DNA shotgun sequencing and RNA-sequencing revealed the dominance of *M. fructigena* followed by *S. borealis*, *S. sclerotiorum*, *B. cinerea*, *Glomus cerebriforme*, *Aspergillus steynii*, *Cryphonectria parasitica*, *Trichoderma atroviride*, and *Talaromyces islandicus*. In particular, this is the first report of *M. fructigena* in peach fruits in Korea. Moreover, we found high amount of proteobacteria including *Frateuria aurantia*, *Neoasaia chiangmaiensis*, *Robbsia andropogonis*, and *Ewingella Americana*. Our results revealed co-inhabitance of fungi and bacteria in the mummified peach fruits although dominant microorganisms were present. Most important things that much of fungal and bacterial genes are actively transcribed. In addition, we identified several viruses and viroids. Bacteriophages were identified by DNA- and RNA-sequencing while viruses and viroids with RNA genomes were identified by only RNA-sequencing. Moreover, we identified a novel mycovirus referred as Monilinia umbra-like virus 1 (MULV1) infecting Monilinia species. Comparative analyses between DNA and RNA sequencing suggest that RNA sequencing provides better detailed information for microbial communities; however, combining DNA and RNA sequencing results increased the diversity of microorganism including fungi, bacteria, viruses, and viroids. In addition, we demonstrated the importance of databases and analysis tools for microbiome study. Taken together, this is the first study reporting the microbial communities on the mummified peach fruits. Our study provides a comprehensive overview of microbial communities in the mummified peach fruits by DNA shotgun sequencing and RNA sequencing.

## Figures and Tables

**Table 1:** Summary of identified plant viruses, viroids, and mycoviruses from three RNA libraries.

			R1		R4		R7	
Virus	Accession No.	Size	Coverage	Reads	Coverage	Reads	Coverage	Reads
Apple chlorotic leaf spot virus	NC_001409.1	7555	0	0	0.3273	36	0	0
Hop stunt viroid	NC_001351.1	302	0.5364	2	19.3444	68	0	0
Peach latent mosaic viroid	NC_003636.1	337	7.5905	30	8.095	35	1.9822	8
Asian prunus virus 2	NC_028868.1	9362	0.0092	1	0.9509	110	0	0
Asian prunus virus 3	NC_028975.1	9654	0	0	0.1859	28	0	0
Monilinia umbra-like virus	MT681745	4147	29.8676	1272	117.3161	4904	0.2358	10

Name, accession number (No.), genome size, coverage, and number of viral reads (Reads) of identified viruses and viroids were provided.

## Supplementary Materials

The following are available online at https://www.mdpi.com/2223-7747/9/8/1052/s1. Table S1: The number of reads associated with fungi in six different libraries, Table S2: The number of reads associated with bacteria in six different libraries, Table S3: The number of reads associated with viruses in six different libraries, Figure S1: Identification of microorganisms from the D4 and R4 libraries using KRAKEN 2 program, Figure S2: Identification of microorganisms from the D7 and R7 libraries using KRAKEN 2 program.
