# Supplementary figures and images for: Comparative Microbiome Study of Mummified Peach Fruits by Metagenomics and Metatranscriptomics
Source: Plants (Basel). 2020 Aug 18;9(8):1052. doi: 10.3390/plants9081052 (PMC7464454; doi:10.3390/plants9081052)

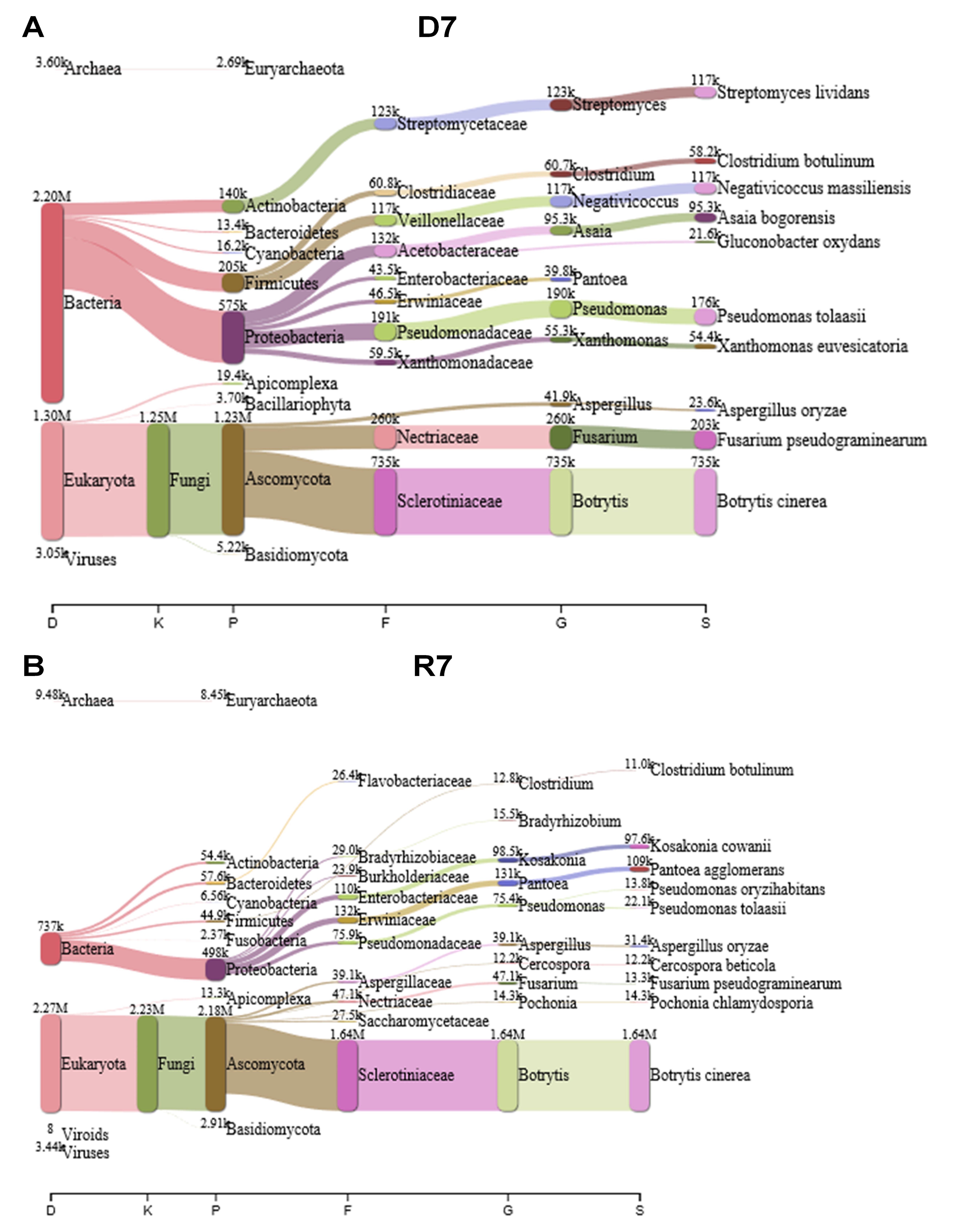

Supplement: Supplementary file 1 [file plants-09-01052-s001.zip › FigureS2.jpg]

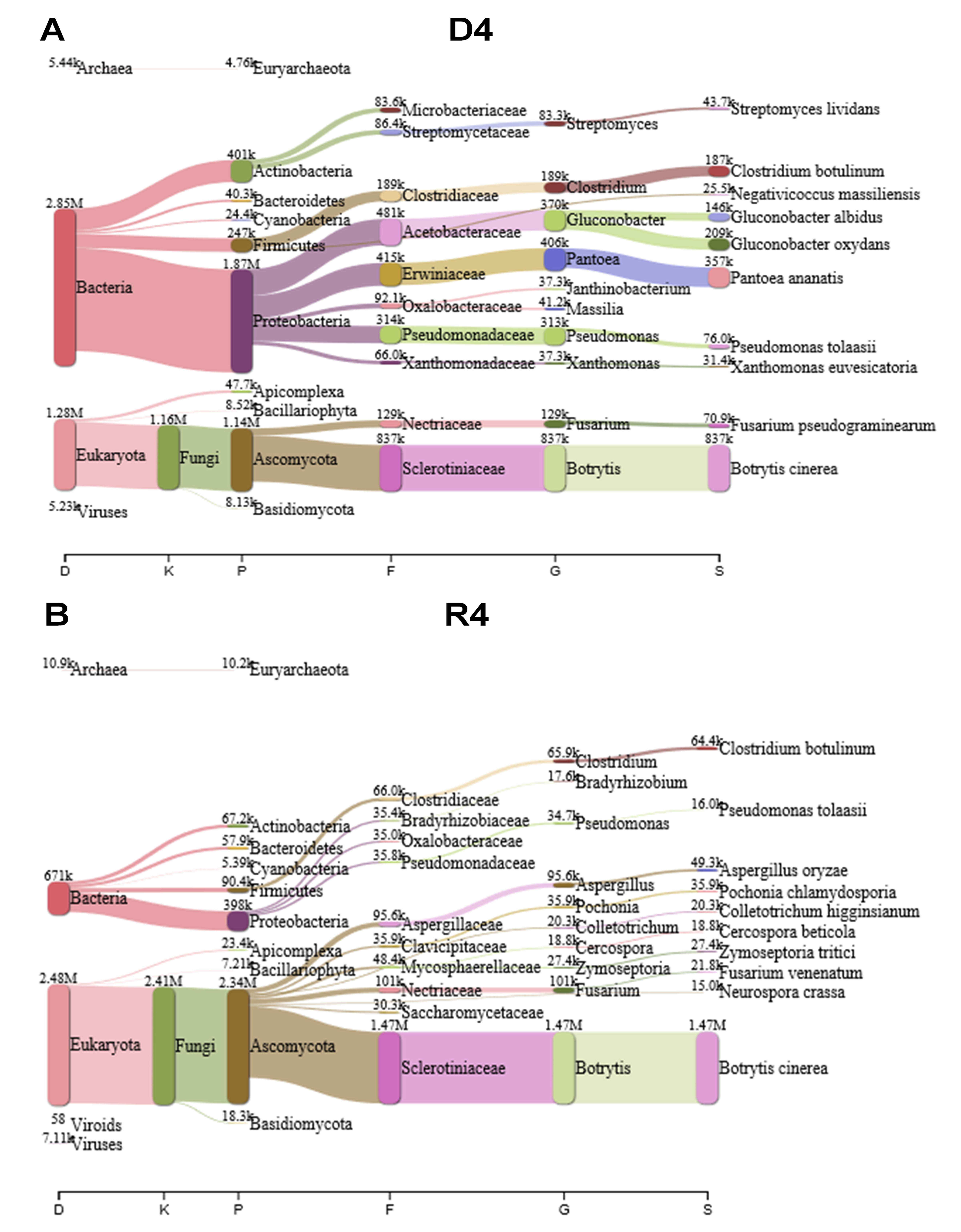

Supplement: Supplementary file 1 [file plants-09-01052-s001.zip › FigureS1.jpg]
